# Scaling-Out Digitally Enabled Integrated Care in Europe Through Good Practices Transfer: The JADECARE Study

**DOI:** 10.5334/ijic.8605

**Published:** 2024-08-09

**Authors:** Ane Fullaondo, Yhasmine Hamu, Jon Txarramendieta, Esteban de Manuel

**Affiliations:** 1 Biosistemak Institute for Health Systems Research, Basque Country, Spain; 2 Network for Research on Chronicity, Primary Care, and Health Promotion (RICAPPS), Spain; 3 Osakidetza, Gorliz Hospital, Basque Country, Spain

**Keywords:** integrated care, digital innovation, healthcare transformation, context, adaptation

## Abstract

**Introduction:**

The absence of a coordinated approach to health and social care compromises the ability of health systems to provide universal, equitable, high-quality, and financially sustainable care. Transferring evidence-based practices focused on digitally-enabled integrated care to new contexts can overcome this challenge if implementation is satisfactory. This paper presents the scaling-out methodology that JADECARE has designed to spread effective innovative practices across Europe.

**Methodology:**

The scaling-out methodology pretends to guide the Next Adopters in the transfer and adoption of practices, whereas increasing their implementation capacity and providing an evaluation framework to assess impact and success.

**Discussion:**

JADECARE scaling-out effort is based on guiding principles found in the literature such as the balance between fidelity to the original practice and the degree of adaptation required to fit the new context, the need for capacity building in implementation to bridge the gap between research and routine practice and the focus on explaining why, for whom and in what circumstances an intervention works.

**Conclusion:**

The JADECARE scaling-out methodology is theory-driven and pragmatic and aims to facilitate the transfer of complex interventions across different contexts.

## Introduction

The ageing of the population with the growing burden of chronic conditions and multimorbidity is steadily increasing the demand for a more extended and efficient and intelligent outcome-based personalized care [1]. Unfortunately, many of the existing European healthcare models focus primarily on short- and medium-term interventions for single conditions [2]. They often overlook the interconnections between different chronic diseases and fail to integrate the care of multiple providers involved [3].

The absence of a coordinated approach to health and social care increases difficulties in aligning care across teams and settings. This seriously compromises the ability of health systems to provide universal, equitable, high-quality, and financially sustainable care [4]. Increased specialization with fragmented care approaches lead to poor communication and information sharing, causing shortcomings and gaps in the care provided to patients with chronic conditions and long-term care needs [5].

The existing evidence suggests that developing integrated person-centred care services should generate significant improvements in the care and health of all citizens (enhanced quality and access to care, better health and clinical outcomes, increased health literacy and self-care) [6]. The overall costs would be reduced as well [7]. Person- centred care identifies common health concerns and needs, shared health objectives and healthcare goals, and appropriate healthcare activities, associated with the healthcare process [8].

The process of transformation towards integrated care requires the combination of a top-down approach, creating the regulatory, financial and governance conditions for change; with a local and emergent bottom-up approach, facilitating teams of professionals and municipalities to implement the national framework according to local needs [9]. Although integrated care as a public policy feature is present in many countries, health systems have acknowledged difficulties in implementing and scaling it up [10].

Innovative solutions based on citizen’s needs through new technologies, products, and organizational changes are needed. Digital innovation, including e-Health, is used worldwide to facilitate and support the delivery of integrated person-centred services [11,12]. Yet, the extension of e-health has often been slower than expected, mainly because of difficulties related with implementation. Findings suggest that issues around implementations are multi-level and complex, no single factor is identified as key barrier or facilitator. The importance of policies and incentives, adequate infrastructure and resources, engagement of key personnel, organizational readiness, individuals’ knowledge and beliefs, and the fit of interventions with workflows, processes and systems is clearly stated in the literature [13]. Harvesting the full benefits of digitally enabled integrated person-centred care demands that digital health tools are embedded into healthcare and social care delivery systems.

The journey of care delivery transformation in Europe is still in its first stages. The systematic incorporation of evidence-based interventions into real practice and policy can improve healthcare performance and outcomes [14]; population-wide health improvements, though, depend on large-scale implementation of effective health interventions. The transfer and spread of proven effective innovative practices to new contexts is a possibility of great interest. However, a gap remains between the development of evidence-based public health interventions and their successful implementation [15]. Transferring complex intervention is a challenging endeavour, due to the number of components involved; the range of behaviours targeted; expertise and skills required, the number of persons, groups, settings, or levels targeted; or the contexts in which the interventions operate. Moreover, when an evidence-based intervention is implemented with fidelity in a setting that is very similar to the context wherein it was previously found to be effective, it is reasonable to anticipate similar benefits of original intervention. However, this is not entirely true when transferring programs and policies into a novel scenario or targeting distinct populations. Therefore, adaptation of interventions [16], intentional changes to improve the contextual fit within a new scenario, is crucial if similar benefits are expected in the adopter scenario [17,18] and it proves to be more efficient than de novo intervention development [19].

Additionally, regarding the implementation of integrated care, there are some specific challenges that should be considered, since integrated care is not a simple approach to adopt. First, health and social care systems are extremely complex with multiple organizations, structures, financial models, cultures, professional groups, and legal responsibilities. Second, there is high propensity of health and social care settings for service development and change, requiring solid staff engagement and time to embed new practices. Third, non-linear distribution of status, power and resources between sectors that are supposed to work collaboratively, and the recognition of professional identity by focusing on individuals’ strengths and skills as opposed to asserting a generic approach. And last, the communication of vision and expectations, so everyone is aligned and in agreement on shared outcomes [20].

In this challenging context, the European Commission launched a series of initiatives to support countries in health promotion and prevention of non-communicable diseases within the 3rd Health Programme (2014–2020). One of such initiatives is the **J**oint **A**ction on implementation of **D**igitally **E**nabled integrated person-centred **care** (JADECARE) [21]. This Joint Action aims to reinforce the capacity of health authorities to successfully address important aspects of health system transformation, in particular the transition to digitally enabled, integrated, person-centred care. JADECARE pretends to transfer four evidence-based Good Practices selected by the Steering Group on Health Promotion and Prevention and Management of Non-Communicable Diseases (SGPP) of the European Commission, from original healthcare systems (Early Adopters) to other healthcare systems (Next Adopters). The four Good Practices are: Basque Health strategy in ageing and chronicity (Basque Country, Spain), Catalan Open Innovation Hub on ICT-supported Integrated Care Services for Chronic Patients (Catalonia, Spain), the OptiMedis Model-Population-based integrated care (Germany), and the Digital roadmap towards an integrated health care sector (Southern Denmark). JADECARE involves 45 organizations from 16 European countries (Belgium, Croatia, Czech Republic, Denmark, Estonia, France, Germany, Greece, Hungary, Italy, Latvia, Portugal, Serbia, Slovenia, Spain, and the United Kingdom).

The JADECARE original Good Practices involve complex and resource intensive strategies comprising several features related to integrated care, health risk stratification, patient empowerment and healthcare regulation. Transferring complex interventions to new contexts is a demanding process that profoundly changes the basic routines, resource and authority flows, as well as beliefs of the social system in which the innovation is implemented [22]. This paper presents the scaling-out methodology that JADECARE has designed to overcome these challenges.

## Original Good Practices

JADECARE is focused on the transfer and adoption of four Good Practices that were selected by the Steering Group on Health Promotion and Prevention and Management on Non-Communicable Diseases in February 2019 [23]. These Good Practices were chosen amongst 10 best practices in the field of digitally enabled, integrated, person-centred care, coming from EU initiatives and programmes such as the European Innovation Partnership on Active and Healthy Ageing and projects financed by the Health Programme. All the proposals were show-cased and assessed by the SGPP on 12–13 December 2018 at the Joint Research Centre (JRC) in Ispra (Italy) [24].

The four Good Practices were selected according to the set of criteria defined by the SGPP: relevance, intervention characteristics, evidence and theory based, ethical aspects, effectiveness and efficiency, equity, transferability, sustainability, participation and intersectoral collaboration [25].

The main area addressed by the Good Practices is the integrated care, not only within the healthcare settings but also across health and social care environments. Target populations approached are people with chronic conditions (e.g. hypertension, diabetes, COPD, asthma, heart failure), patients with multimorbidity and with complex needs, and frail people, whereas the scope of practices encompasses population health, disease management and case management.

### Basque health strategy in ageing and chronicity – integrated care (Basque Country)

Is a prevention-driven and population-based approach focused on risk stratification, digitally enabled integrated care and patient/citizen empowerment. It places particular emphasis on care planning, new organizational pathways, continuity of care, remodelling of professional roles, incorporation of digital tools, data analytic for decision-making support, and patient training for self-management.

### Catalan Open Innovation Hub on ICT-Supported Integrated Care Services for Chronic Patients (Catalonia)

Encompasses both vertical (hospital and community-based care) and horizontal (healthcare and social support) integrations. It combines a population-health orientation with a collaborative adaptive case management approach of specific integrated care services. Synergies among relevant stakeholders of the health and social care system are promoted, guaranteeing the healthcare continuum with support of digital tools, and complementing the individual approach with a population-based perspective.

### The OptiMedis Model – Population based integrated care (Germany)

Is based on the creation of territorial networks of health and non-health services providers coordinated by an independent local integrator. Its main goal is to produce health through patient centred services, case and care management, the development of mutually beneficial relationships and the establishment of incentive systems to reward integrated interventions. Digital solutions facilitate better target setting, patient risk stratification allow personalized care planning, business intelligence services strengthen care networks and enable population, patient, and provider outcome measurement.

### Digital roadmap towards an integrated health care sector (Southern Denmark)

Pretends to improve and strengthen the existing cooperation between the healthcare sectors. The roadmap consists of different elements that together set up the foundation for digital and cross-sectorial communication. This is based on a strong collaboration between the different organizations in the regional ecosystem of academia, knowledge institutions and private companies. Focus is on stakeholder involvement of both professionals and end-users in co-designing solutions, implementation of processes and the development of a strong information technology infrastructure to make digital communication possible.

The Good Practices will be transferred to 21 Next Adopters from 14 European countries (Belgium, Croatia, Czech Republic, Denmark, Estonia, France, Greece, Hungary, Italy, Latvia, Portugal, Serbia, Slovenia, and Spain).

## Description of the methodology

**Figure 1:**
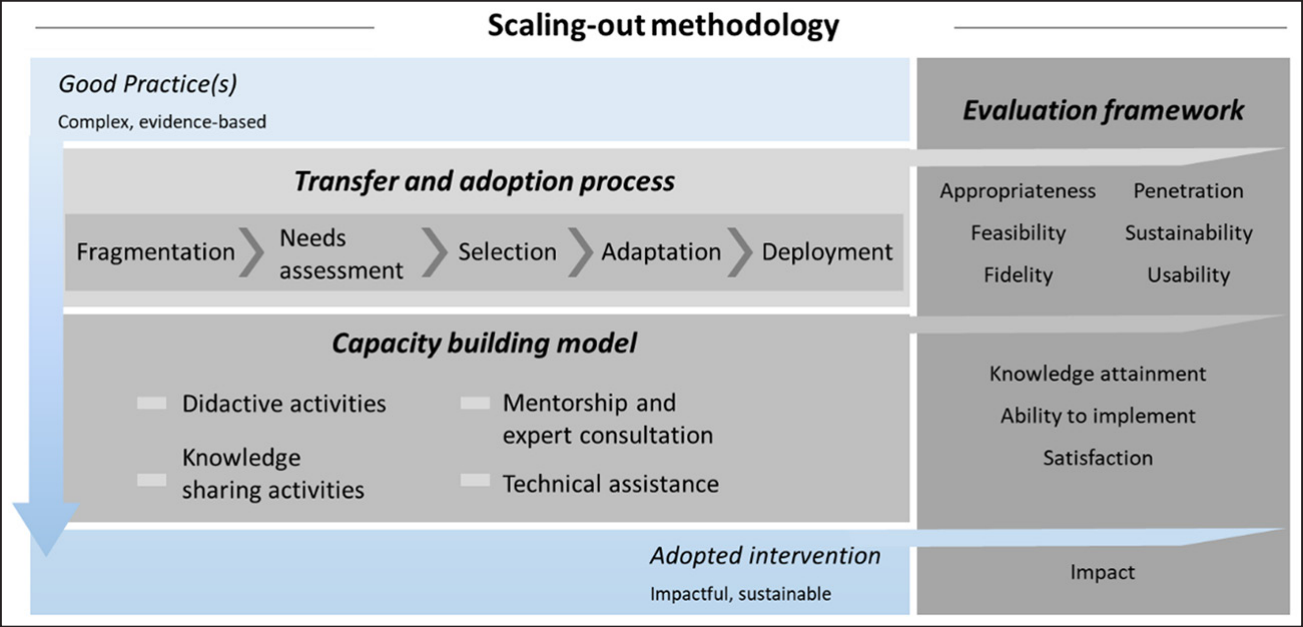
Scaling-out methodology.

The scaling-out methodology has been devised to guide and support Next Adopters in designing and implementing their interventions according to their real needs and possibilities/resources. The methodology developed is grounded on the interconnection of independent theoretical models that address crucial aspects when it comes to the implementation of complex interventions in disparate contexts. The Consolidated Framework for Implementation Research [26] and the ADAPT guidance [27] are key pillars of the methodology presented in this study. Both models not only specify the critical issues to be considered during the implementation process (transfer and adoption mechanisms), but also emphasize the relevance of analysing the readiness of the organizations, implementation facilitators and support delivered, and evaluation of the effectiveness of the interventions. Building on this knowledge, the scaling-out methodology includes three interrelated elements: the Transfer and adoption process, the Capacity building model, and the Evaluation Framework (Figure 1).

### Transfer and adoption process

The transfer and adoption process consists of four steps that will guide the scaling-out of the Good Practices to develop the adapted interventions.

1. *Fragmentation*. The breakdown of each complex Good Practice into smaller and more easily transferable components is essential if it is intended to support Next Adopters on selecting the elements that they can implement within the constraints of the project (limited duration and funding). Thus, the Good Practices will be fragmented by Early Adopters into thematic Core Features (CF), that is, the meaningful units that represent the minimum level to which Good Practices can be unpacked while maintaining sufficient substance to produce the expected impact on health [28].
2. *Needs assessment*. In JADECARE healthcare needs of Next Adopters will be assessed. Healthcare needs can be covered by services that are supplied by healthcare providers, including health education, disease prevention, diagnosis, treatment, rehabilitation, and terminal care [29]. In JADECARE a corporate approach [30] is proposed which involves the systematic collection of the knowledge and views about healthcare services and needs of key informants (health authority staff, managers, clinicians, general practitioners, nurses, community health councils, as well as users). From the operational perspective, the Next Adopters will form a multidisciplinary implementation team including these key informants and they will participate in a workshop to assess healthcare needs at local level by means of the following questions: (1) What the problem is; (2) What the size and nature of the problem are; (3) What services are currently offered; and (4) What patients want.
3. *Selection*. Based on the outputs of the previous steps, the fragmentation of the Good Practices and the formative research resulting in the identification of healthcare needs at Next Adopter level, a correlation matrix will be constructed. It will establish a mapping of the Core Features of the Good Practices and the local needs of Next Adopters, specifying which needs are met and by which feature(s). Then, a co-creation approach (the process of involving stakeholders in the development of the services and interventions to foster sharing and discussion around possible ways of addressing a specific issue [31]) will be followed by Next Adopters. The correlation matrix will be presented to the multidisciplinary team of each Next Adopter, in such a way that they can evaluate whether the Core Features associated to their needs are appropriate and a priority [32]. The assessment will be performed using a methodology inspired in the multi-criteria decision analysis [33], including dimensions such as acceptability, fidelity, feasibility, scalability, and sustainability. This can be summarized as the implementability of the intervention [34]. This assessment will seek to capitalize on the strengths of the system and the opportunities provided by the environment, while minimizing the impact of the various threats. As a result of this process, the Next Adopters will select the Core Features, either only from one Good Practice or from various Good Practices. These features will delimit the scope of the intervention to be implemented along the Joint Action JADECARE, covering their local needs, and aligned with their interests.
4. *Adaptation*. The sum of the Core Features selected by the Next Adopters will construct the Adopted Interventions. These interventions are nourished by components coming from the Good Practices, as explained in the previous step, that already have demonstrated to be effective. The Adopted Interventions will be adapted (incorporation of intentional modifications to the original practice) to achieve better fit with the new context [18,35]. Implementation teams of Next Adopters will perform the following activities: (i) exploration of the selected Core Features, (ii) identification of potential mismatches between Core Features and the context, (iii) undertaking modifications (intervention content, language, or delivery mechanisms), and (iv) Adopted Intervention development.
5. *Deployment*. The Adopted Interventions will be rolled-out in the new contexts based on Quality Improvement models that propose developing change actions, designing multiple tests of increasing complexity, collecting, and analysing data and making appropriate updates to the change actions [36]. Next Adopters will implement actions that lead to the roll-out of the whole intervention in a cyclical fashion using small tests of change before taking changes system-wide [37].

### Capacity building model

Capacity building interventions will help to increase the competencies of the Next Adopters’ teams to move their Adopted Interventions past the pilot phase and support spread and scaling-up efforts. The following activities will be deployed:

- *Didactic activities*. Specific *thematic webinars* will be scheduled and will be led by implementation science experts to provide guide during the different steps of the Good Practice transfer and adoption process.
- *Mentorship and expert consultation*. At the beginning of JADECARE *study visits* will be planned where Early Adopters present the details of the Good Practices including potential barriers that can hinder transferability to other contexts. During the implementation, *thematic workshops* will be held to bring visibility to Next Adopters at their local/regional/national level and boost communication and cooperation with key stakeholders. These workshops will provide a valuable opportunity to assess the progress of the implementation as well as to collaboratively reflect on solutions to overcome existing challenges. Additionally, representatives of the Good Practices (Basque Country, Catalonia, Germany, and Southern Denmark) will be available for consultation when needed.
- *Knowledge sharing activities*. At the end of the transfer and adoption process *implementation key learning workshops* will be held to encourage networking, reflection, and the discussion of implementation experiences among the Next Adopters.
- *Technical assistance*. A thorough guide of the methodology will be developed. Moreover, experts on implementation science will provide continuous support (email, phone) to the Next Adopters mainly focused on techniques of the scaling-out methodology.

### Evaluation framework

#### Effectiveness and usability of the transfer and adoption process

The effectiveness of the JADECARE transfer and adoption process in achieving implementation outcomes will be evaluated as an indicator of the implementation success. The dimensions investigated will be:

- Appropriateness: perceived fit, benefit, relevance, or compatibility of evidence-based practice for a given practice setting, provider, or consumer.
- Feasibility: the extent to which a new practice can be successfully used or carried out within a given setting.
- Fidelity: the degree to which an intervention or practice was implemented as it was conceived originally or as it was intended by the practice developers.
- Penetration: the integration of a practice within a service setting and its subsystems.
- Sustainability: the extent to which the implemented practice is maintained or institutionalized within a service setting’s ongoing, stable operations.

In addition, the usability [38] of the transfer and adoption process will be assessed. Next Adopters’ multidisciplinary teams will provide feedback in terms of the structure, content, and complexity of the approach.

Quantitative and qualitative measures will be used to assess both the effectiveness and usability of the transfer and adoption process by means of a survey to be completed by Next Adopters, who will follow the proposed steps (fragmentation, needs assessment, selection, adaptation, and deployment).

#### Power of the capacity building model

In JADECARE, members of the multidisciplinary team of Next Adopters (healthcare professionals, managers, and policy makers) are the targets of the implementation practice capacity building interventions. Expected outcomes can be classified into the following categories:

- Knowledge attainment: Understanding and awareness of implementation models, factors and strategies influencing evidence use.
- Perceived ability to implement evidence: Participants’ ability to implement evidence into practice, as measured through self-efficacy, confidence, and competence.
- Satisfaction: Perceived acceptability, appropriateness, or approval of capacity building intervention structure and content.

The assessment of these outcomes will be based on a mixed approach (formative and summative evaluations) that will be performed by means of surveys provided to Next Adopters’ teams at different time-points during the implementation process. Knowledge attainment and perceived ability to implement Adopted Interventions will be studied at the end of the process, whereas the satisfaction with capacity building interventions will be explored both after the precise mentorship and knowledge sharing activities (study visits, thematic workshops, and implementation key learning workshops) as well as at the end of the complete transfer and adoption experience.

#### Impact of the Adopted Interventions

The assessment of the impact of the implemented interventions is based on a realistic evaluation approach, which pretends to uncover the mechanisms that lead to observed outcomes following an intervention and the contextual conditions in the Next Adopters. The focus is on explaining why, for whom and in what circumstances an intervention works. It is a theory-driven approach and both quantitative and qualitative data collection methods can be used to unearth the underlying mechanisms that cause the intervention outcomes [39]. The realist evaluation in JADECARE is based on the survey method targeting key stakeholders of the Next Adopters (convenience sampling) which utilizes both self-created surveys to elicit views on the different elements of the scaling-out methodology (the power of the capacity building model and the impact of the adopted interventions) and standardized and validated tests (for the usability assessment of the transfer and adoption process guidelines). Collected data from surveys will be presented in a descriptive way [39].

The areas to be explored are compiled in the framework on integrated people-centred health services published by the World Health Organization [40]. This document encompasses broad areas where integrated care policies and interventions can have an impact:

- Empowering and engaging people and communities: empower individuals to make effective decisions about their own health and to enable communities to become actively engaged in co-producing healthy environments, and to provide informal carers with the necessary education to optimize their performance.
- Strengthening governance and accountability: policy formulation, mutual accountability, decision-making, and performance evaluation at all levels of the health system, from policymaking to the clinical intervention level.
- Reorienting the model of care: encompasses the shift from inpatient to outpatient and ambulatory care, and from curative to preventive care, including health promotion and ill-health prevention strategies, and incorporating new technologies.
- Coordinating services within and across sectors: integration of health care providers within and across health care settings, development of referral systems and networks among levels of care, and the creation of linkages between health and other sectors.
- Creating an enabling environment: involve a diverse set of processes to bring about the necessary changes in leadership and management, information systems, methods to improve quality, reorientation of the workforce, legislative frameworks, financial arrangements, and incentives.

## Discussion

This work presents the scaling-out methodology to promote the replication of Good Practices (complex interventions consisting of several components, defined as being the parts that make the whole intervention and, in isolation or combination, can generate the power of the intervention [41]) across European countries to enhance the transition of healthcare systems to digitally enabled, integrated, person-centred care. Scaling-out is a concept used in different fields (computer science [42], social innovation [43], economics) and implies replicating successful interventions with adaptation to new contexts via cogeneration of knowledge [44]. JADECARE efforts to implement an evidence-based intervention within a new context consists of different steps: (1) local needs identification and context analysis; (2) definition of the adapted local Good Practice; (3) local practice implementation and monitoring, and (4) process and outcome assessment.

The purpose of needs assessment in health care is to gather the information required to bring about changes beneficial to the health of the population. It is not simply a process of listening to patients or relying on personal experience. It is a systematic method of identifying unmet health and healthcare needs of a population and making changes to respond to these unmet needs (what should be done, what can be done, and what can be afforded) [45].

Flexibility in intervention delivery and adherence might be permitted to allow for variation in how, where, and by whom interventions are delivered and received. Standardisation of interventions could relate more to the underlying process and functions of the intervention than on the specific form of components delivered [46]. Therefore, in alignment with the proposal in JADECARE, interventions require a theoretical fragmentation into components and then agreement about permissible and prohibited variation in the delivery of those components. This allows implementation of a complex intervention to vary across different contexts yet maintain the integrity of the core components, aspects of an intervention that cannot be modified [47,48]. Fidelity is concerned with adherence to these central activities, while peripheral elements can be modified [49].

Historically, adaptation has largely focused on the modification of intervention components and delivery strategies. Emerging complex system perspectives have theorised that interventions are inseparable from the contexts in which they operate [50,51]; however, limited attention has been paid to the need to modify aspects of the new context to accommodate the intervention. It is crucial to consider the interactions between the intervention and its context (understood as any feature of the circumstances in which an intervention is conceived, developed, implemented and evaluated) to increase the likelihood of transfer success [28,49,52]. The methodology presented in this article, which combines contributions of different theoretical perspectives such as complex systems thinking, implementation science and realistic evaluation, is rooted in the context-wise concept, understanding that interventions require adaptation primarily when there are mismatches between contexts [27].

Facilitation constitutes one of many implementation approaches used to support change within organizations. Several studies, most of which relate to practice facilitation and knowledge translation, reveal various forms of facilitation, with focuses ranging from achieving specific goals (task) to developing processes for better teamwork (holistic) [53]. There is a battery of facilitation activities described in the literature that can be divided into four groupings: planning for change, leading and managing change, monitoring progress and ongoing implementation, and evaluating [54]. The JADECARE scaling-out methodology is designed on the principles behind these activities, which seek for increasing awareness of actual needs, recognizing the importance of the context, fostering team building, providing mentoring and guidance, and boosting networking. The JADECARE capacity building model recognizes and addresses the necessity of enhancing individual, organisation or system capabilities to conduct and implement high-quality research and practice [55,56]. Empowering key stakeholders to implement evidence in routine practice and policy settings is crucial [55], to reduce the implementation research-to-practice gap [57].

The JADECARE realist approach proposed for the evaluation of the three related elements of the scaling-out methodology (transfer and adoption process, capacity building model and impact of the interventions) is based on Context-Mechanism-Outcome Configuration [58], which depicts the central form of causal reasoning emphasizing the relevance of the environment. It has been shown that realist evaluation is appropriate when complex interventions are implemented in rapidly changing environments (such as healthcare service delivery) and there are many unpredictable forces that determine the programme’s (integrated care interventions) scope and architecture, as well as resultant outcome [59,60].

However, this approach presents some limitations. The survey method chosen might encounter difficulties to capture the causal structures and mechanisms behind the outcomes since quantitative approaches often lack interpretative depth and probably might need adaption to realist evaluation paradigm [61]. Innovative qualitative research methods such as realist Q methodology [62], photovoice [63], soft systems methodology [64] and causal loop diagramming [65] might be options to be considered. Regarding the sampling, a purposeful method might be a better approach to avoid selection bias and ensure the involvement of different perspectives close to the implementation of the digitally enabled integrated care interventions.

## Conclusion

The JADECARE scaling-out methodology provides a process model and guidance to facilitate the transfer and adoption of complex evidence-based interventions across different contexts, addressing specific challenges related to the adaptation of the interventions, empowerment of key stakeholders involved in the implementation and the complexity of the outcome assessment framework. This methodology is theory-driven and pretends to be usable and pragmatic. The aim is that it should be efficient and low burden, feasible to conduct in real-world settings, and yield actionable information that directly informs decisions about implementation [66].
